# Time series and trend analysis of brucellosis in Oskou county, East Azerbaijan: 2007-2016

**DOI:** 10.15171/hpp.2019.39

**Published:** 2019-10-24

**Authors:** Hosein Rafiemanesh, Yousef Alimohamadi, Seyed Rasoul Hashemi Aghdam, Avaz Safarzadeh, Abolghasem Shokri, Alireza Zemestani

**Affiliations:** ^1^Student Research Committee, Department of Epidemiology, School of Public Health and Safety, Shahid Beheshti University of Medical Sciences, Tehran, Iran; ^2^Noor Research Center for Ophthalmic Epidemiology, Noor Eye Hospital, Tehran, Iran; ^3^Department of Epidemiology, School of Public Health, Tehran University of Medical Sciences, Tehran, Iran; ^4^Oskou Comprehensive and Public Health Network, Tabriz University of Medical Sciences, Tabriz, Iran; ^5^Student Research Committee, Urmia University of Medical Sciences, Urmia, Iran; ^6^Department of Epidemiology, School of Public Health, Shahid Beheshti University of Medical Sciences, Tehran, Iran; ^7^Road Traffic Injury Research Center, Health Management and Safety Promotion Research Institute, Tabriz University of Medical Sciences, Tabriz, Iran

**Keywords:** Brucellosis, Incidence, Epidemiology, Time series analysis, Oskou, Iran

## Abstract

**Background:** The epidemiology of human brucellosis has drastically changed in recent years. This study aims to assess trend in brucellosis in the Oskou county, East Azerbaijan, Iran.

**Methods:** This cross-sectional study was conducted on all confirmed brucellosis cases over the period between 2007 and 2016 in Oskuo county. We use crude incidence rate (CIR) per100000 persons and carried out Joinpoint regression analysis to describe brucellosis trend over the study period. Also, we used ARIMA model to predict trend and number of new brucellosis cases for the coming years.

**Results:** More than 90% (92.5%; 95% CI: 89.9-95.1) of brucellosis cases were in rural areas over the study period. In recorded cases, 60.5% (95% CI: 55.6-65.4) of total cases were men and 39.5% (95% CI: 34.6-44.4) of total cases were women. The mean age of men was 33.85(SD=19.72) years and the mean age of women was 35.88 (SD=17.26) years old. Majority of brucellosis cases occurred in spring. CIRs for the rural and urban areas were 47.62 to132.20 and zero to 18.55, respectively. The CIR for rural area had decreasing trend to 2011 and increasing for 2011-2017.

**Conclusion:** Based-on time series analysis, the number of new cases in the future years has fixed trend and the most number of incident cases will be occurred between third to fifth months in each years.

## Introduction

Brucellosis or Malta fever is a common bacterial systemic disease between humans and animals, and is sometimes transmitted directly or indirectly through contact with an infected animal.^1^ This disease is a severe and/or acute illness characterized by persistent, intermittent or irregular fever symptoms, headaches, weakness, sweating, joint pain, depression, weight loss, splenomegaly, and myalgia in the patients. It is caused by different types of bacteria Brucella.^1-3^ Brucellosis is mainly a work-related disease and is seen in those who work with contaminated animals or their tissues. The disease is more common in men than women. The source of contamination and infection is different in each geographic region.^1^

While the incidence of human brucellosis varies 0.3 to 1603.4 per million population across the countries, its incidence in Iran is 238.6.^4^ Brucellosis is an endemic disease in Iran, which in most provinces comes to more than 34 people per 100 000 people.^5^ The disease is rapidly increasing in some Middle East countries including Saudi Arabia, Iran, Palestine, Syria, Oman and Jordan. Iran is considered one of the highest ranked countries in terms of the incidence of Brucellosis in the world.^6^

Brucellosis is a disease with serious economic consequences and may take several days, months and sometimes several years to recover. As widespread disease in the world, brucellosis is a public health concern and reduce productivity, abortion and weakness in the livestock. It is the cause of significant economic losses in livestock production due to reproductive disorders and reduced production of affected animals.^1,4^

The various sanitary, socioeconomic and political reasons, along with the development of air travel, have caused the brucellosis epidemiology to change in the world.^4^ The geographical distribution of brucellosis is constantly changing, with new foci emerging or re-emerging.^7^ The epidemiology of human brucellosis has drastically changed over the past few years because of various sanitary, socioeconomic, and political reasons, together with increased international travel.^4^

According to studies conducted by the Ministry of Health and Medical Education (MoHME) of Iran, East Azerbaijan is one of the most prevalent provinces in terms of brucellosis in the country.^5^ Considering the fact that the Oskou County also has a high prevalence of brucellosis, this study aimed to study the epidemiological pattern and estimate the ten-year trend of brucellosis in this County using the data of the communicable diseases surveillance system.

## Materials and Methods

The present study is a cross-sectional study that was conducted on all individuals with brucellosis who were identified by the governmental and non-governmental sectors in the period from 2007 to 2016 in Oskou county in the brucellosis surveillance system (BSS). In the BSS, all brucellosis cases were reported from health centers, hospitals and laboratories in the governmental and non-governmental sectors, as well as physician offices. After confirmation of the cases and completion of an epidemiologic form by physicians or experts, the brucellosis cases were sent to the Prevention and Control of Diseases Unit (PCDU) in the county’s health center. Analyses were conducted on all confirmed cases of brucellosis registered in the Oskou county over the period of ten years. All of the information in epidemiologic forms were confidential in this study, and patients’ information was analyzed by encoding without mentioning the patients’ names.

### Statistical analysis

We calculated crude incidence rate (CIR) per 100 000 persons. To describe brucellosis trend in ten years, we use CIR and carried out Joinpoint regression analysis using the Joinpoint software, version 4.5.0.1 (National Cancer Institute, Bethesda, MD).

Joinpoint regression analysis involves fitting a series of joined straight lines on a log scale to the trends. The approach aims to identify possible Joinpoints where a significant change in the trend occurs.^8^ The final selected model was the most parsimonious model, with the estimated annual percent change (APC) based on the trend within each segment. In describing trends, the terms “significant increase” or “significant decrease” indicates that the slope of the trend was statistically significant (*P* < 0.05). All reported *P* values were two-sided.

After performing stationary transformations for variance by using Box-Cox procedure and mean by differentiation, the best model for time series analysis selected according to partial autocorrelation function (PACF) and autocorrelation function (ACF) graphs for determining autoregressive (AR(p) (and moving average (MA)q) (with minimum Akaike information criterion (AIC). After selection of best models for prediction of data, the selected model assessed for fitness by using tests of randomness on residuals such as Ljung–Box statistic, McLeod-Li statistic, turning points, difference sign points, rank test statistic, Jarque-Bera test statistic (for normality) and the schematic Checking of the residual graph. All analysis performed by ITSM (version 2000), Stata 14 (College Station, TX: StataCorp LLC) and Microsoft Excel 2016 software Microsoft, United States) with regard α < 0.05 for significant level.

## Results

During March 2007 to March 2017, the total number of brucellosis that were recorded in the county was about 385 cases. The mean age of cases was about 34.65 (SD = 18.79) years. In recorded cases 233 (60.5%) of total cases were men and 152 (39.5%) of total cases were women. The mean age of men was 33.85 (SD = 19.72) years and the mean age of women was 35.88 (SD = 17.26) years old. 75 (19.48%) of cases were reported in Ilkhchi city, 304 (78.96%) of cases were reported in Osku and 6 (1.56%) of cases were reported in Sahand city. 356 (92.47%) of total cases was reported in rural area and 29 (7.53%) of cases was reported in urban area. Seasonally, the most cases were occurred in the spring (39.5%) and summer (30.9%) seasons.

### Trend analysis

More than 75% of the cases were occurred in rural areas. The CIR (per 100 000 population) for the rural and urban areas were between 47.62 to 132.20 and zero to 18.55, respectively (Table 1).

Join point trend analysis showed a decreasing trend for the CIR for total county with APC of -7.4 (95% CI: -15.9 to 1.9; *P* = 0.1) in 2008-2017. The CIR for rural area had two not significant trends; decreasing for 2008-2011 period with annual percentage change (APC) = -27.9 95% CI: -56.3 to 18.9; *P* = 0.2) and increasing for 2011-2017 period with APC = 7.4 (95% CI: -9.3 to 27.1; *P* = 0.3).

**Figure 1:**
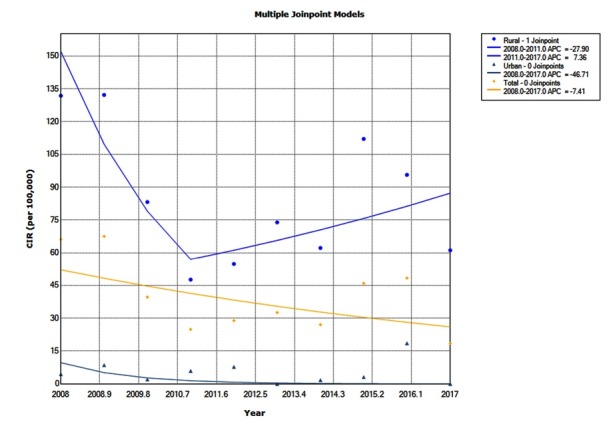
Joinpoint analysis of brucellosis crude incidence rate (CIR) in Oskou County, East Azerbaijan, Iran, 2007-2016.

Trend for urban area (assuming a crude incidence equal to 0.0001 per 100 000 for zero years) was decreasing with APC = -46.7 (-82.8 to 65.1; *P* = 0.2) in 2008-2017 (see Figure 1).

### Time series analysis

**Figure 2:**
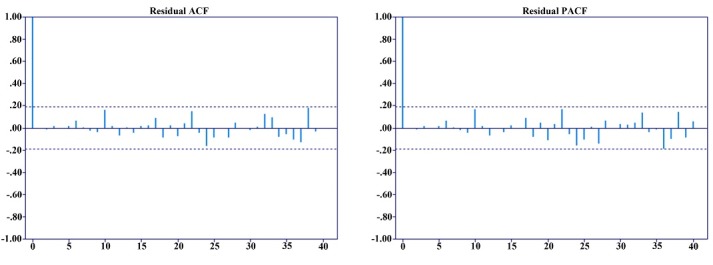
Residual graph of ACF and PACF for assessing fitness of selected model.

**Figure 3:**
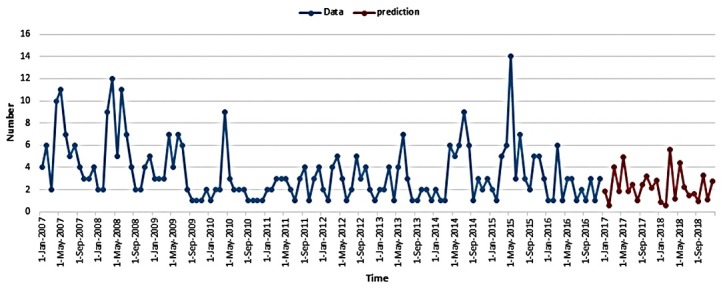
Time series and prediction of incident brucellosis trend for coming years in Oskou County, East Azerbaijan, Iran, 2007-2016.

To select the best model of prediction, we used PACF and ACF models and minimum AIC statistic. We used ARIMA model with AR: 14, MA: 4 and AIC = 402 (ARIMA14, 1, 4) to predict trend and number of new brucellosis cases for the coming years. The tests of randomness on residuals was shown in Table 2. According to the table, the selected model for prediction on the number of incident brucellosis cases is appropriate. The graph of residuals was shown in Figure 2. The prediction of new brucellosis cases has been presented in Figure 3. According to this table, the number of new cases in the future years has fixed trend and the most number of incident cases will be occurred between third to fifth months in each years (Figure 3).

## Discussion

Increasing brucellosis in the human population reflects the spread of disease in the animal population. This necessitates the control of disease in livestock population. Brucellosis can occur in all age group, but the most common age groups are young people and adolescents and economically active population.^9-12^ In this study, the disease mainly affected the most productive group in the Oskou county, East Azerbaijan, Iran. These results clearly show how the age range reflects the magnitude of the socioeconomic and cultural impact of brucellosis in the study region. The mean age of the patients was about 35 years old. This finding is also confirmed in other similar studies.^13,14^

The results of this study showed that the number of new cases for 10 years in men was 1.5 times higher than women. Brucellosis is primarily a an occupational disease and is more common in men.^15^ Considering that most of this study population were rural residents and their jobs are livestock workers, so the high incidence of disease in men can be attributed to their job. Some studies have shown that the number of brucellosis cases is equal in men and women.^16-18^ For example, in the study by Buzgan et al in Turkey of the total of 1028 cases, 539 (52.4%) were women and 489 (47.6%) were women.^14^ This inconsistency in the results could be due to differences in the demographic characteristics of studies. In many studies, livestock population density is known as a risk factor for brucellosis in livestock.^19-21^ Several studies in the world have been confirmed the link between animal and human contamination.^22^

This study showed a downward trend in the incidence of brucellosis reported in the Oskou county over the study period (2007 to 2016). Similar studies in countries such as Saudi Arabia and Iran suggested a decrease in the incidence of brucellosis in recent years, which is consistent with the findings of this study.^9-13^ Studies have shown that the risk of human disease in the world has declined due to vaccination of livestock.^23,24^ Thus, the overall decreasing trend shown in the present study could be due to increased coverage of prevention programs, especially the vaccination of livestock. The results of the present study showed an increasing trend in CIR in rural region after 2001. This can be due to increased sensitivity of communicable diseases surveillance system to detect and record the disease.

The results of time series analysis showed the number of new cases in the future years has a fixed trend and the most cases of incident cases will be between third to fifth months in each year. Considering that this period is a breeding season for sheep, it seems that due to the contact and exposure of ranchers and breeders to delivery discharge and placenta of sheep and to breathe in the contaminated space this season, one of the reasons for the increase in disease is this season.

### Limitations

1-Incomplete information of some patients in epidemiological forms, which fortunately in our study was less than 2%.

2- The questionnaire and the epidemiological survey form were not comprehensive so that information such as type of clinical symptoms, type of pathogen, and type of livestock, number of livestock and exact vaccination status of animals were not collected.

3- At the time of the study, complete and clean data were only available until year 2016, so we conducted the study to that date.

### Strengths

1. Higher sensitivity of the laboratory surveillance system to identify cases of brucellosis
2. Desired cooperation of physicians, health workers and health professionals in completing the epidemiological examination forms.

### Implications for practice and policy making

1. Using the results of this study to estimate the resources of the surveillance system such as human resources, finances, hospital beds and planning to provide these resources
2. Using the results of this study to prevent the disease.

### Future directions

Considering the tendency of people to use dairy products in the traditional way and the reluctance to use pasteurized dairy products, it is recommended to educate people on the use of pasteurized dairy products in order to prevent the increasing prevalence of the disease.

## Conclusion

The most cases of brucellosis are in the men and young age group and most of cases was occurred in rural areas. According to results, the number of new cases in the future years has a fixed trend in the Oskou county, East Azerbaijan, Iran and most number of incident cases will be between third to fifth months in each year.

## Ethical approval

All epidemiological forms after entering the computer were analyzed by their assigned code and patient information was kept confidential.

## Competing interests

The authors declare that they have no competing interests.

## Funding

This study was not funded by any organization.

## Authors’contributions

All authors contributed to writing the manuscript. AZ and HR have made substantial contributions to conception and design. SRHA, AvS, AbS have made contributions to review the epidemiological forms and data entry. HR, YA and AZ have been made contributions to dada analyses. HR, YA, AZ and SRHA have been made substantial contributions to critically review the manuscript for important intellectual content, and have given final approval of the version to be published.

## Acknowledgments

The authors sincerely thank all the physicians and supervising educators of the health centers as well as the workers of health houses (Behvarz) of Oskou Comprehensive and Universal Health Network who have contributed to collecting study data and completing epidemiological survey forms over the past years.

**Table 1:** Frequency and crude incidence rate (CIR) of brucellosis in Oskou County, East Azerbaijan, Iran, 2007-2016

Year*	**Rural(n=356)**		**Urban(n=29)**		**Total(n=385)**	
	**No.(%)**	**CIR**	**No.(%)**	**CIR**	**N(100%)**	**CIR**
2007-2008	55 (96.5)	131.83	2 (3.5)	4.50	57	66.13
2008-2009	56 (93.3)	132.20	4 (6.7)	8.59	60	67.47
2009-2010	35 (97.2)	83.16	1 (2.8)	2.05	36	39.65
2010-2011	20 (87.0)	47.62	3 (13.0)	5.95	23	24.90
2011-2012	23 (85.2)	54.78	4 (14.8)	7.79	27	28.92
2012-2013	31 (100)	73.89	0.0	0.00	31	32.61
2013-2014	26 (96.3)	62.19	1 (3.7)	1.73	27	27.08
2014-2015	46 (95.8)	111.99	2 (4.2)	3.15	48	45.93
2015-2016	39 (76.5)	95.57	12 (23.5)	18.55	51	48.34
2016-2017	25 (100)	61.14	0.0	0.00	25	18.47

*21 March X (year) to 20 March X+1.

**Table 2:** Results of different tests of randomness on residuals in number of incident brucellosis cases

**Tests of randomness on residuals**	**Value of each statistics**	***P*** **value**
(2)Ljung - Box statistic	18.84, chi-square (20)	0.532
McLeod - Li statistic	54.26, chi-square (29)	0.003
# Turning points	71.0~AN (70.67, SD= 4.34)	0.939
# Diff sign points	53.0~AN (53.50, SD= 3.014)	0.868
Rank test statistic	0.31590E+04~AN (0.28890E+04, SD =0.18834E+03)	0.152
Jarque-Bera test statistic (for normality)	0.0811, chi-square (2)	0.960
Order of Min AICC YW model for residuals	0	-
